# A rank-based transcriptional signature for predicting relapse risk of stage II colorectal cancer identified with proper data sources

**DOI:** 10.18632/oncotarget.7956

**Published:** 2016-03-07

**Authors:** Wenyuan Zhao, Beibei Chen, Xin Guo, Ruiping Wang, Zhiqiang Chang, Yu Dong, Kai Song, Wen Wang, Lishuang Qi, Yunyan Gu, Chenguang Wang, Da Yang, Zheng Guo

**Affiliations:** ^1^ College of Bioinformatics Science and Technology, Harbin Medical University, Harbin, China; ^2^ Key Laboratory of Ministry of Education for Gastrointestinal Cancer, Department of Bioinformatics, Fujian Medical University, Fuzhou, China; ^3^ Department of Pharmaceutical Sciences, University of Pittsburgh, Pittsburgh, PA, USA; ^4^ Women's Cancer Research Center, University of Pittsburgh Cancer Institute, Pittsburgh, PA, USA; ^5^ Department of Computational & Systems Biology, University of Pittsburgh, Pittsburgh, PA, USA

**Keywords:** gene expression profiles, prognostic signatures, gene pairs, experimental batch effect, relative expression

## Abstract

The irreproducibility problem seriously hinders the studies on transcriptional signatures for predicting relapse risk of early stage colorectal cancer (CRC) patients. Through reviewing recently published 34 literatures for the development of CRC prognostic signatures based on gene expression profiles, we revealed a surprising phenomenon that 33 of these studies analyzed CRC samples with and without adjuvant chemotherapy together in the training and/or validation datasets. This data misuse problem could be partially attributed to the unclear and incomplete data annotation in public data sources. Furthermore, all the signatures proposed by these studies were based on risk scores summarized from gene expression levels, which are sensitive to experimental batch effects and risk compositions of the samples analyzed together. To avoid the above-mentioned problems, we carefully selected three qualified large datasets to develop and validate a signature consisting of three pairs of genes. The within-sample relative expression orderings of these gene pairs could robustly predict relapse risk of stage II CRC samples assessed in different laboratories. The transcriptional and functional analyses provided clear evidence that the high risk patients predicted by the proposed signature represent patients with micro-metastases.

## INTRODUCTION

Colorectal cancer (CRC) is the third most common cancer and the fourth leading cause of cancer death worldwide [1]. The main factor for therapeutic decisions and prognostic estimates is based on AJCC tumor stage. Patients with stage II disease used to be treated with surgery only. However, retrospective analyses on historical trials show that approximately 25-30% of stage II CRC patients undergoing curative surgery will experience relapse, and only these patients need adjuvant CTX to reduce the relapse risk [2, 3]. Currently used clinical and pathologic parameters, such as intestinal perforation/obstruction, tumor size and tumor grade [4], molecular markers, such as mutations in KRAS and BRAF as well as chromosome and microsatellite instability (MSI) [5-10] cannot adequately assess relapse risk to guide the clinical adjuvant CTX after surgery [11, 12].

High-throughput gene expression profiling has emerged as a powerful tool to identify stage II CRC patients with potential relapse risk [13, 14]. However, previously reported prognostic transcriptional signatures often fail to be validated in independent datasets [15-18]. Therefore, it is necessary to analyze the major factors, besides the commonly claimed problem of small sample sizes [18], that may lead to the irreproducibility of the reported prognostic signatures. A surprising phenomenon is that most of current studies for the development of CRC prognostic signatures based on gene expression profiles have the data misuse problem, which could be partially attributed to the unclear and incomplete data annotation in public data sources (see Results). Besides, most of current gene expression signatures, including those signatures based on functional categories [19-21], are based on risk scores calculated as some summaries of expression levels of signature genes, which are sensitive to experimental batch effects and could lead to irreproducibility of prognostic signatures [22]. For the applications of this type of prognostic signatures, the requirement of presetting risk score thresholds and data normalization would result in the risk classification of patients depends on the risk composition of the samples adopted for normalization together [22]. This could produce substantial uncertainty for patient risk classification especially when the sample sizes are insufficient to represent the disease populations [22]. It has been revealed that within-sample relative expression orderings (REOs) of genes are overwhelmingly stable in a particular type of normal human tissues, which could reflect the concerted correlations of gene expression in normal states, but widely disrupted in the corresponding cancer tissues [23]. This biological phenomenon provides a basis for analyses based on REOs of gene pairs to characterize cancer subtypes [24-26]. Because REOs of genes are insensitive to experimental batch effects of gene expression profiling and invariant to monotonic data normalization [27], it is worthwhile to apply the rank-based approach to find robust prognostic signatures for clinical application.

**Figure 1:**
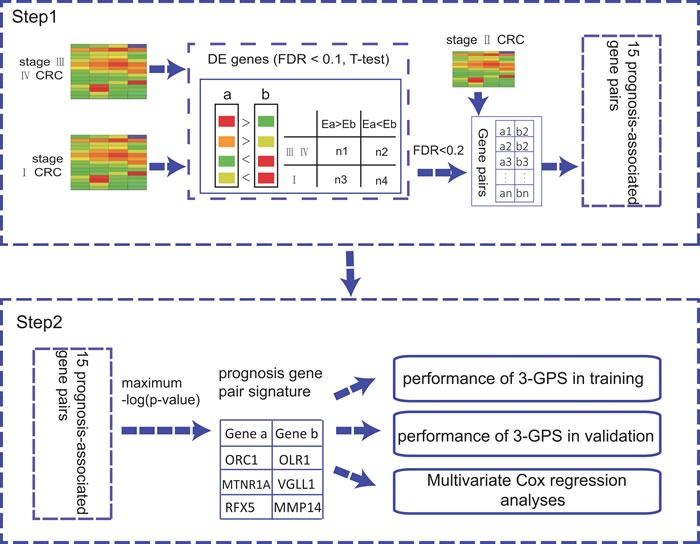
The flowchart for the development of the rank-based prognostic GPS

In this work (shown in the Figure 1), firstly, we showed that different CRC sample sets have heterogeneous risk compositions, which could produce substantial uncertainty for patient risk classification based on risk scores summarized from gene expression levels. Then, we extracted a rank-based prognostic signature for relapse risk of stage II CRC using three large datasets.

## RESULTS

### Problems of data usage and data annotation of public data sources

By searching the PubMed medical literature database, we collected a total of 34 studies published in English between January 1, 2010, and June 1, 2015 for developing prognostic signatures of CRC based on gene expression profiles (shown in Table 1). Surprisingly, except for the study of ColoGuideEx [14], all the other 33 studies used a mixture of samples with and without CTX in the training and/or validation datasets, although it has been well recognized that pooling cancer samples with and without CTX for prognostic signatures discovery is unreasonable as adjuvant CTX could affect the relapse-free survival (RFS) of the patients [28, 29]. This surprising phenomenon of data misuse could be partially attributed to incomplete and inaccurate clinical data annotation of CRC samples collected in public databases. First, the clinical annotations of many datasets are incomplete. For example, the dataset GSE17536 in the GEO database [30] provides no information indicating whether the collected samples are for patients treated with CTX or not. Some researchers used this dataset for identifying the relapse risk signature, wrongly considering that the samples in this dataset after surgery did not undergo CTX without discriminating patients [31]. However, we found that 42 stage III CRC patients' samples documented in this dataset did accept CTX by tracing the original research papers [32, 33]. Second, some samples are repeatedly documented in different datasets. For example, 35 stage II CRC samples that were repeatedly measured are documented in two series of the GEO database, GSE14333 and GSE17538, but this information is not provided in the data description [30]. Consequently, these two datasets with technical replicates were used as both the training and validation sets in some studies such as the study for ColoGuidePro [13], resulting in non-independent verification of the signature. Due to these problems, we have to check the original papers to avoid inappropriate use of expression profiles documented in the public data sources.

**Table 1:** Proposed gene expression signatures for prognostic assessment of CRC

Date	Datasets	Mixed	Tumor stage	Prognostic endpoint	References(PubMed index)
2015	GSE12945, GSE41258, GSE14333, GSE17538, GSE29623, GSE33113, GSE39582, GSE24549, GSE24550, GSE30378, GSE28722	yes	I-IV	OS	25853550
2015	GSE17536	yes	I-IV	DSS	25622900
2015	GSE24549, GSE24550, GSE39582	yes	I-IV	OS	25894381
2014	GSE13294, GSE5206, GSE17536, GSE17537	yes	II-III	DFS	24486594
2014	GSE14333, GSE17538, GSE33113, GSE31595, GSE14095, GSE26892	yes	II-III	Relapse	25115384
2014	GSE14333, GSE33113, GSE17538	yes	I-III	Relapse	24829396
2014	GSE17536, GSE17537, GSE38832	yes	II-III	OS, DSS and DFS	25320007
2014	GSE17536, GSE30378	yes	I-IV	DSS	25000257
2014	GSE17538, GSE14333	yes	II-III	RFS	24728738
2014	GSE17538, GSE14333	yes	II-III	RFS, OS	25504183
2014	GSE39582, GSE14333, GSE17536	yes	I-IV	DFS	24809982
2013	GSE14333, GSE17538	yes	I-IV	RFS	23372686
2013	GSE14333, GSE17538, GSE12032	yes	I-IV	DFS, DSS	23658834
2013	GSE17536	yes	I-IV	OS	24247253
2013	GSE17536	yes	I-IV	DSS	23799978
2013	GSE17536, GSE14333	yes	I-III	DFS	22859720
2013	GSE17536, GSE14333	yes	I-IV	OS	23807160
2013	GSE17536, GSE14333, GSE12945	yes	I-IV	OS	24140838
2013	GSE17536, GSE17537	yes	I-III	OS	23922772
2013	GSE17536, the training data was not provided	yes	II-III	OS	24170546
2013	GSE17537	yes	I-IV	RFS	24360964
2013	GSE17538	yes	I -IV	OS	24052018
2013	GSE17538, GSE14333, GSE37892	yes	I-IV	DFS	23626670
2012	GSE12032, GSE17538, GSE17181, GSE4526	yes	II-III	Relapse	22348113
2012	GSE14333, GSE17538, GSE30378, GSE24550	yes	II-III	RFS	22991413
2012	GSE17536, GSE14333	yes	I-IV	RFS	22710688
2012	GSE17536, GSE14333	yes	I-III	DFS	22859720
2012	GSE17536, GSE14333	yes	II-III	RFS	22844451
2012	GSE17537, GSE14333	yes	I-IV	RFS	23153532
2012	GSE29638, GSE24550, GSE30378	no	II	RFS	22213796
2011	GSE5206, GSE10402	yes	I-IV	RFS	21098318
2011	GSE5206, GSE17537	yes	I-IV	OS	22977525
2010	GSE17538	yes	II-III	OS, RFS	19914252
2010	GSE17538, GSE14333	yes	II-III	DFS, DSS	21119668

**Abbreviations: RFS,** relapse-free survival, also called **DFS,** disease-free survival. **OS,** overall survival. **DSS,** disease specific survival.

### Heterogeneous risk compositions of independent datasets

**Figure 2:**
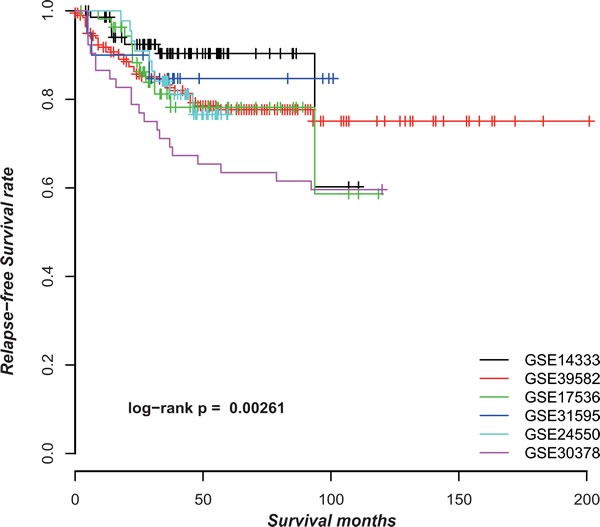
The Kaplan-Meier curves of RFS for samples in six datasets

CRC is clinically and pathologically highly heterogeneous with a large variation in 5-year survival rates in different countries and even different cities in the same country [1]. As shown in Figure 2, the stage II CRC patients without CTX after surgery from six datasets (Supplementary Table S1) had significantly different RFS (*p* = 0.0026, log-rank test). The heterogeneous risk compositions could be due to many factors such as the differences in diagnosis criteria, surgery quality and location of CRC. For signatures based on risk scores summarized from gene expression measurements of a set of signature genes, this problem would induce spurious risk classification and difficulty in clinical settings because the risk classification of a sample would change when different samples are adopted for analysis together [25].

For example, ColoGuideEx [14], a 13-gene prognostic classifier, assigned patients to a poor prognosis group when at least 5 genes in the 13-gene signature indicated poor prognosis. If the high or low expression of a gene included in the 13-gene signature was associated with the high risk of relapse, and its expression level in a sample was above the 80th or below the 20th percentile of its expression levels among all the samples, then it was considered to indicate poor prognosis for this particular sample [14]. As the 80th and 20th percentile of a gene's expression values in a set of samples are dependent on the samples analyzed together, the risk classification of a sample by ColoGuideEx may change when it is analyzed together with different samples. We analyzed the 52 stage II CRC samples of the GSE30378 dataset to illustrate this problem. ColoGuideEx classified 45 of the 52 samples into the low-risk group. Applying ColoGuideEx to reanalyze these 45 low-risk samples, 8 samples were reclassified into the high risk group, indicating the uncertainty of this classifier for the risk classification of patients [22].

### The gene pair signature for the relapse risk of stage II CRC

We used the GSE39582 (*n* = 203) dataset with the largest sample size to train a GPS of the relapse risk for stage II CRC and validated it in the GSE14333 and GSE17536 datasets (shown in the Table 2). Because GSE17536 (*n* = 55) included 35 samples, which were technical replicates of the samples of GSE14333, we considered it as a validation dataset for technical reproducibility of the signature.

**Table 2:** The CRC datasets used in this work generated on GPL570 platform

Dataset	Stage I CRC#	Stage II CRC# without CTX	Stage II CRC# with CTX	Stage III CRC#	StageIV CRC#
GSE39582	33	203	56	205	60
GSE14333	44	72	22	91	61
GSE17536	24	55	0	57	39

**Figure 3:**
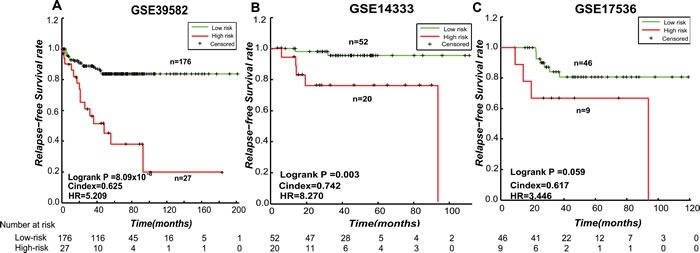
The Kaplan-Meier curves of RFS for stage II CRC samples stratified by the 3-GPS in the training and validation datasets **A.** The training dataset GSE39582; **B.** The independent validation dataset GSE14333; **C.** The validation dataset GSE1736. A sample was classified into high-risk group (red line) only if at least two gene pairs in the 3-GPS voted for high-risk.

Based on the hypothesis that the stage II CRC at high risk of relapse could be attributed to micro-metastasis, we firstly extracted 174 and 278 Metastatic-DE genes (Student's *t*-test, FDR < 0.1) from the GSE39582 and GSE14333 datasets, respectively, and then found 149472 and 1154605 significantly reversed gene pairs (Fisher exact test, FDR < 0.2), each consisting at least one of the Metastatic-DE genes, between the metastatic samples (stage III and IV) and the non-metastatic samples (stage I) in the two datasets, respectively. The two lists of gene pairs had 6386 overlaps and 99.86% of them had the same reversal patterns in the two datasets, which was unlikely to be observed by chance (*p* < 2.2 × 10^−16^, the binomial distribution model). Finally, from the 6377 metastasis-associated gene pairs consistently detected in the two datasets, we extracted 15 prognosis-associated gene pairs based on 203 stage II CRC samples from GSE39582 dataset, by univariate Cox proportional-hazards regression model with *p* < 0.01. The 15 prognosis-associated gene pairs are listed in Supplementary Table S2. Among these 15 prognosis-associated gene pairs, using the gene pair ORC1-OLR1 with the smallest log-rank *p*-value as a seed, we performed a forward selection procedure and obtained an optimal set consisting of three gene pairs reached the smallest *p*-value (C-index = 0.625, log-rank *p* = 8.09 × 10^−8^, HR = 5.209, shown in Figure 3A). We selected these three gene pairs as the final prognostic signature, referred to as 3-GPS (ORC1-OLR1, MTNR1A-VGLL1 and RFX5-MMP14 shown in Supplementary Table S3). For each of the three gene pairs, the *E*a < *E*b REO was associated with worse survival. Thus, a simple rule was used to classify patients: a sample was classified into the high-risk group only if at least two gene pairs suggested that this sample was at high risk. A multivariate Cox analysis showed that, after adjusting for age, gender, MSI and localization, the 3-GPS remained significantly associated with patient RFS (log-rank *p* = 7.28 × 10^−6^, HR = 7.5479, 95% CI, 3.121-18.257, shown in Table 3).

**Table 3:** Multivariate Cox regression analyses of the 3-GPS

Clinical Characteristic	HR	Cox *p* value	95% CI
GPS(High Risk *vs* Low Risk)	7.5479	7.28 × 10^−6^	[3.121, 18.257]
Age	2.7269	0.0423	[1.034, 7.182]
Sex (Male *vs* Femal)	0.9944	0.7477	[0.961, 1.029]
Localization (distal vs proximal)	1.9770	0.1794	[0.731, 5.348]
MSI	0.6845	0.6922	[0.105, 4.471]
Braf mut	3.8353	0.2086	[0.472, 31.178]
Kras mut	0.8843	0.8322	[0.284, 2.757]
Tp53 mut	1.2155	0.6301	[0.549, 2.690]

The 3-GPS was validated in the stage II CRC samples without CTX included in the GSE14333 (*n* = 72) dataset. The RFS of the patients in the low-risk groups was significantly longer than that of the patients in the high-risk group (Figure 3B, C-index = 0.7424, log-rank *p* = 0.003, HR = 8.270). The 3-GPS was also validated in the stage II CRC samples without CTX included in the GSE17536 (*n* = 55) dataset (Figure 3C, C-index = 0.618, log-rank *p* = 0.059, HR = 3.446).

### 3-GPS as a micro-metastatic signature

Using the Student's *t*-test with 10% FDR control, we extracted DE genes between the high- and low-risk groups identified from the training and validation datasets, denoted as Risk-DE genes, respectively. We found 98.09-100% of the Risk-DE genes commonly detected in any two of the three datasets were consistent in dysregulation directions in the high-risk group compared with the low-risk group, which was unlikely to happen by chance (binomial distribution test, *p* < 2.2 × 10^−16^, shown in Table 4). This result proved that the 3-GPS could robustly categorize stage II CRC patients into the high- and low-risk groups with distinct transcriptional characteristics. Finally, we obtained 1003 Risk-DE genes by integrating the Risk-DE genes from the three datasets according to the following criterion: DE genes selected in at least two of the three datasets were included in the list, after excluding those DE genes that had inconsistent dysregulation directions in any two datasets.

**Table 4:** The consistency of the Risk-DE genes detected from three datasets

Dataset1	Dataset2	DE genes 1	DE genes2	overlap	consistency
GSE39582	GSE14333	3599	2540	836	98.09%
GSE39582	GSE17536	3599	505	364	99.45%
GSE14333	GSE17536	2540	505	247	100%

Moreover, for each of the three datasets, Risk-DE genes were significantly overlapped with the Metastatic-DE genes detected between the stage III-IV samples and stage I samples (*p* = 0.0307 for GSE39582, *p* < 2.2 × 10^−16^ for GSE14333 and *p* = 6.79 × 10^−10^ for GSE17536, hypergeometric distribution model, shown in Table 5). For the overlapped Risk-DE genes and Metastatic-DE genes, all the dysregulation directions in the high-risk samples compared with the low-risk samples had concordant dysregulation directions in the metastatic samples compared with the non-metastatic samples (*p* = 4.55 × 10^−13^ for GSE39582, *p* < 2.2 × 10^−16^ for GSE14333 and *p* = 2.4 × 10^−4^ for GSE17536, binomial distribution test). Functional enrichment analysis showed that the 1003 Risk-DE genes were significantly enriched in six KEGG pathways (FDR < 0.05, hypergeometric distribution, shown in Table 6). Five of these six pathways are well-known metastasis-associated pathways, including “ECM-receptor interaction” [35], “Focal adhesion” [36], “PI3K-Akt signaling pathway” [37-39], “Glycosaminoglycan biosynthesis-chondroitin sulfate/dermatan sulfate” [40] and “Regulation of actin cytoskeleton” [41]. The sixth pathway, “Protein digestion and absorption” [42], has also been reported to be associated with CRC development [42]. These results provided evidence that the high risk patients predicted from the stage II CRC patients by the 3-GPS represented patients with micro- metastases.

**Table 5:** The consistency between the Risk-DE genes and the Metastatic-DE genes

Dataset1	Risk-DE genes#	Metastatic -DE genes#	Overlap	p_1_	Consistency	p_2_
GSE39582	3599	174	41	0.0307	100%	4.55 × 10^−13^
GSE14333	2540	278	118	<2.2 × 10^−16^	100%	<2.2 × 10^−16^
GSE17536	505	45	12	6.79 × 10^−10^	100%	2.4 × 10^−4^

Notes: #, the number of Risk-DE genes and Metastatic-DE genes; *p*_1_, the *p* value of overlaps between Risk-DE genes and Metastatic-DE genes; *p*_1_, the *p* value of the concordance score of the overlapped DE genes.

**Table 6:** The KEGG function enrichment analysis results

Pathway name	Adjusted *p*-values	References(PubMed index)
ECM-receptor interaction	2.22 × 10^−14^	9854310
Focal adhesion	7.99 × 10^−10^	15246682
Protein digestion and absorption	4.97 × 10^−5^	21490305
PI3K-Akt signaling pathway	2.98 × 10^−3^	7558426
Glycosaminoglycan biosynthesis-chondroitin sulfate/dermatan sulfate	5.29 × 10^−3^	24035453
Regulation of actin cytoskeleton	4.38 × 10^−2^	11709869

### 3-GPS risk stratification for potential benefit from CTX

The GSE39582 and GSE14333 datasets also included 56 and 22 samples of stage II CRC patients treated with CTX (shown in Table 7), besides the 203 and 72 samples of stage II CRC patients without CTX treatment, respectively. For each of the dataset, we used the 3-GPS signature to divide all stage II CRC samples with and without CTX into the high- and low-risk groups and compared the RFS between samples with and without CTX in the high- and low-risk groups, respectively.

**Table 7:** The distribution of stage II CRC predicted by the 3-GPS

Datasets	L-risk CRC with CTX	L-risk CRC without CTX	H-risk CRC with CTX	H-risk CRC without CTX
GSE39582	46	176	10	27
GSE14333	11	52	11	20
All	57	228	21	47

Abbreviations: CTX, the patients with completely resected tumors who received adjuvant chemotherapy. L-risk or H-risk represents the patients with low or high relapse risk.

**Figure 4:**
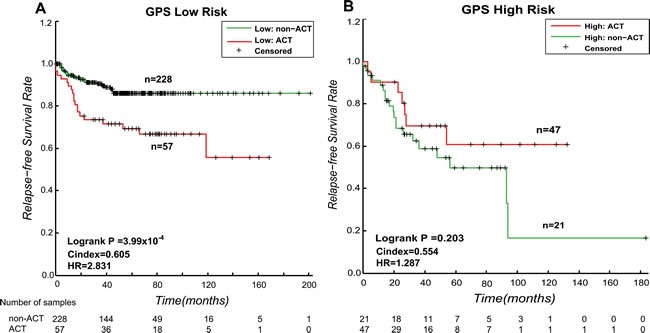
Kaplan-Meier estimates of the RFS of GSE39582 and GSE14333 patients with CTX and non-CTX patients **A.** Kaplan-Meier curves for stage II CRC patients in the low relapse risk group. **B.** Kaplan-Meier curves for stage II CRC patients in the high relapse risk group.

Due to the small size of the stage II CRC patients with adjuvant CTX, we integrated the samples in these two datasets. The 3-GPS signature assigned 68 (21 samples with CTX and 47 samples without CTX) and 285 (57 samples with CTX and 228 samples without CTX) patients to the high- and low-risk groups, respectively. For the patients predicted to the low relapse risk group, the RFS of the patients with CTX was significantly shorter than that of the patients without CTX (Figure 4A, C-index = 0.6049, log-rank *p* = 3.99 × 10^−4^, HR = 2.831), indicating that the CTX was not beneficial to patients in the low risk group and even shortened their RFS. In contrast, for the patients predicted to the high relapse risk group, the patients with CTX tended to have significantly longer RFS than the patients without CTX (Figure 4B, C-index = 0.5536, log-rank *p* = 0.203, HR = 1.827), indicating that the CTX tended to increase the RFS of patients at high relapse risk.

## DISCUSSION

As shown in this study, most of current studies for the development of CRC prognostic signatures have the data misuse problem and the public data sources need improvement with clear and complete data annotation. Previously reported risk-score based transcriptional signatures tend produce spurious risk classification due to the requirement of data normalization to tackle experimental batch effects [22]. To address this problem, we developed a robust gene pair signature for predicting relapse risk of stage II CRC based on the within-sample REOs of gene pairs. The rank-based signature is robust against batch effects and data normalizations of gene expression profiling experiments and can be easily applied to samples at the individual level [24, 27] and thus it merits further validation in clinical trial.

In this work, we proposed a hypothesis that the stage II CRC patients at high risk of relapse could be largely attributed to micro-metastases, given that most curative resection surgeries for stage II CRC patients are successful (i.e., no cancer cells are remained after surgeries) and that the random chance of a person to develop cancer ab initio is negligible. Our results supported this hypothesis, as evidenced by the observation the Risk-DE genes between the predicted high- and low-risk samples significantly overlapped with the Metastatic-DE genes detected between the metastatic samples (the stage III-IV samples) and non-metastatic samples (stage I samples) and the deregulation directions of the overlapped DE genes in the high-risk samples compared with the low-risk samples were significantly concordant with the deregulation directions in the metastatic samples compared with the non-metastatic samples. Additionally, the Risk-DE genes were significantly enriched in six well-known metastasis-associated pathways, including “ECM-receptor interaction” [35], “Focal adhesion” [36], “PI3K-Akt signaling pathway” [37-39], “Glycosaminoglycan biosynthesis-chondroitin sulfate/dermatan sulfate” [40], “Regulation of actin cytoskeleton” [41] and “Protein digestion and absorption” [42].

Currently, adjuvant chemotherapy (CTX) is the most common therapeutic regimen prescribed for patients with stage III CRC after surgical resection [43], while its routine use in patients with stage II CRC remains controversial [4, 44]. Our result showed that CTX may reduce RFS of those stage II CRC patients predicted into the low-risk group by 3-GPS. This indicated that the predicted low-risk patients would be indeed at the low risk and thus could not benefit but suffer injury from excessive CTX treatment.

## MATERIALS AND METHODS

### Selection of gene expression-based prognostic studies

We searched the PubMed medical literature database (http://www.ncbi.nlm.nih.gov/pubmed/) to identify articles on the analyses of gene expression data for developing prognostic signatures of CRC, published in English between January 1, 2010 and June1, 2015.

### Microarray data and preprocessing

Three large datasets generated on the GPL570 platform were analyzed in this work (Table 2), which separately included at least 50 samples of stage II CRC patients without CTX treatment. The GSE39582 dataset included 56 samples of stage II CRC patients treated with fluorouracil-based CTX (only fluorouracil and folinic acid after surgery), besides the 203 samples of stage II CRC patients without CTX treatment. The GSE14333 dataset included 22 samples of stage II CRC patients treated with fluorouracil-based CTX (either single agent 5-fluouracil/capecitabine or 5-fluouracil and oxaliplatin after surgery) and 72 samples of stage II CRC patients without CTX treatment. All the data were downloaded from the Gene Expression Omnibus database (GEO, http://www.ncbi.nlm.nih.gov/geo/) [30].

The raw data (.CEL files) for each dataset was processed using the RMA algorithm for background adjustment without quantile normalization [45]. Then, each probeset ID was mapped to Entrez gene ID with the custom CDF file. If multiple probesets were mapped to the same gene, the expression value for the gene was summarized as the arithmetic mean of the values of multiple probesets (on the log2 scale).

### Survival analysis

Survival curves were estimated using the Kaplan-Meier method and were compared using the log-rank test [46]. The univariate Cox proportional-hazards regression model was used to evaluate the correlation of gene pairs with the RFS, and the multivariate Cox proportional-hazards regression model was used to evaluate the independent prognostic value of the signature after adjusting for clinical factors including age, gender, stage, MSI status and localization of the tumor (distal or proximal). We adopted the C-index proposed by Harrell et al. [47, 48] to evaluate the overall concordance between the predicted risk classification and the observed RFS.

### Development of the prognostic gene pair signature

Firstly, using Student's *t*-test, we selected differential expression genes between metastatic samples (stage III and IV CRC) and non-metastatic samples (stage I CRC), denoted as Metastatic-DE genes. From gene pairs including at least one Metastatic-DE genes, we selected gene pairs whose REOs were associated with metastasis. Let *E*a and *E*b represent the expression levels of gene a and gene b, respectively, we compared the frequency of samples with the REO pattern *E*a > *E*b between the metastatic CRC samples and the stage I CRC samples using Fisher exact test with 20% FDR control. The *p*-values were adjusted using the Benjamini-Hochberg (BH) procedure [49]. The overlapped gene pairs between the two lists of significant gene pairs identified from GSE39582 and GSE14333 were defined as metastasis-associated gene pairs.

Then, for each of the metastasis-associated gene pairs, we classified stage II CRC samples without CTX into two groups according to the REO of this gene pair in each sample and compared RFS between the two groups using the univariate Cox proportional-hazards regression model. A gene pair was defined as a prognosis-associated gene pair if the two groups of samples had significantly different RFS. If the *E*a > *E*b REO was associated with worse outcome, then we considered that this REO and the reversal REO (*E*a < *E*b) in a cancer sample votes for high and low risk, respectively. Gene pairs with *p* values less than 0.01 were considered as candidate prognosis-associated gene pairs. We chose the gene pair with the smallest *p*-value as a seed and added a prognosis-related gene pair at each iteration until the *p*-value did not decrease based on the classification rule as follows: a sample was classified into the high-risk group if the majority of the REOs of a set of gene pairs within this sample voted for high risk; otherwise, into the low risk group. The optimal set was defined as the gene pair signature (GPS).

The GPS was validated by two independent datasets GSE14333 and GSE17536. Figure 1 describes the flowchart for developing and validating the rank-based GPS for the risk of the relapse on stage II CRC.

### Concordance scores

If two lists of DE genes between the high- and low-risk groups detected separately from two datasets had *k* overlapped genes, among which *s* genes showed the same deregulation directions (up- or down-regulation) in the two DE gene lists, then the concordance score was calculated as *s/k*. The probability of observing a concordance score of *s/k* by chance was evaluated by the cumulative binomial distribution model as following [50]:

$$P=1-\sum_{i=0}^{s-1}\left(\begin{array}{c} k\\ i \end{array}\right){\left(P_{e}\right)}^{i}{\left(1-P_{e}\right)}^{k-1}$$

where *P_e_* is the probability of one gene having the concordant relationship between the two lists of genes by chance (here, *P_e_* = 0.5).

The significance of a score indicated that DE genes extracted from independent datasets were significantly consistent.

### Functional enrichment analysis

The gene categories for functional enrichment analysis were downloaded from KEGG (http://www.genome.jp/kegg/) in July, 2014. The hypergeometric distribution model was used to test whether the number of DE genes annotated in a functional category was significantly more than what expected by random chance:

$$P_{k}=1-\sum_{i=0}^{m-1}\frac{\left(\begin{array}{c} n\\ i \end{array}\right)\left(\begin{array}{c} N-n\\ M-i \end{array}\right)}{\left(\begin{array}{c} N\\ M \end{array}\right)}$$

where *N* is the total number of the measured genes with functional annotation; *n* is the number of DE genes with functional annotation; *M* is the number of the measured genes in a functional category and *m* is the number of the DE genes in the functional category. The *p*-values were adjusted using the Benjamini-Hochberg (BH) procedure [49].

## SUPPLEMENTARY MATERIAL TABLES


