# Supplementary material for: A rank-based transcriptional signature for predicting relapse risk of stage II colorectal cancer identified with proper data sources
Source: Oncotarget. 2016 Mar 7;7(14):19060–71. doi: 10.18632/oncotarget.7956 (PMC4951352; doi:10.18632/oncotarget.7956)
Supplement: Supplementary file 1 [file oncotarget-07-19060-s001.pdf]

A rank-based transcriptional signature for predicting relapse risk of stage II colorectal cancer identified with proper data sources

Supplementary Material

Supplementary Table S1: Description of the six CRC datasets contained stage II CRC without CTX

GEO Accession	Platforms	Stage II CTX CRC#	Stage II no CTX CRC#
GSE24550	GPL5175	--	44
GSE30378	GPL5175	0	52
GSE31595	GPL570	0	20
GSE17536	GPL570	0	57
GSE14333	GPL570	22	72
GSE39582	GPL570	56	203

Abbreviations: -- means not available. CTX, the patients with completely resected tumors who received adjuvant chemotherapy. No CTX, the patients with completely resected tumors who did not receive any adjuvant chemotherapy.

Supplementary Table S2: The 15 prognosis-associated gene pairs

Id	Gene a	Gene b
1	ORC1	OLR1
2	FBXO9	MTNR1A
3	RFX5	MMP14
4	MTNR1A	VGLL1
5	DLGAP1-AS2	CHSY3
6	SUV39H2	OLR1
7	FXN	ANGPTL4
8	DANCR	VKORC1
9	SUV39H2	FIBIN
10	TRAF3	COLEC12
11	SLC12A2	AZIN1
12	TROVE2	ZC3H12A
13	GSG2	OLR1
14	LRRC40	THBS3
15	RSPO3	SCAI

Note: all the Ea < Eb REOs was associated with poor patient survival.

Supplementary Table S3: The GSP of the risk of relapse on stage II CRC

**Gene A**	**Gene B**	**Beta coefficients**	**Log-rank *p* value**
ORC1	OLR1	1.355	3.5×10^{-4}
MTNR1A	VGLL1	1.074	7.4×10^{-4}
RFX5	MMP14	1.152	3.7×10^{-4}

Note：all the *E*a < *E*b REOs was associated with poor patient survival.
